# Nurses' self‐esteem, self‐compassion and psychological resilience during COVID‐19 pandemic

**DOI:** 10.1002/nop2.1682

**Published:** 2023-02-22

**Authors:** George Vellaramcheril Joy, Albara Mohammad Ali Alomari, Kalpana Singh, Nesiya Hassan, Kamaruddeen Mannethodi, Jibin Kunjavara, Badriya Al Lenjawi

**Affiliations:** ^1^ Nursing and Midwifery Research Department Hamad Medical Corporation Doha Qatar

**Keywords:** compassion, COVID‐19 pandemic, nurse, resilience, self‐esteem

## Abstract

**Aim:**

This study aimed to identify self‐esteem, self‐compassion and psychological resilience among staff nurses during the COVID‐19 pandemic in Qatar.

**Design:**

Descriptive cross‐sectional survey design.

**Methods:**

The study was conducted on January 2022 (during the third wave in Qatar). Anonymous data were collected through an online survey using Microsoft forms from 300 nurses in 14 health facilities in Qatar. Socio‐demographic information, Connor‐Davidson Resilience Scale, Rosenberg Self‐Esteem Scale and Self‐Compassion Scale‐Short Form were used to collect the data. Correlation, *t*‐test and ANOVA analyses were conducted.

**Results:**

Participants expressed a high level of resilience, self‐esteem and self‐compassion. Resilience scores were positively and significantly correlated with self‐esteem and self‐compassion. The education level of nurses was a statistically significant contributing factor to self‐esteem and resilience.

## 1 INTRODUCTION

The invasion of the novel coronavirus disease quickly overshadowed the international year of the nurses and the aftereffects of COVID‐19 have continued to reverberate around the world (LoGiudice & Bartos, 2021). Nurses are the biggest workforce within healthcare systems and an integral part of the management of COVID‐19 pandemic (Shechter et al., 2020).

Uncertainty was the main challenge to nurses that covered a wide range of concerns including, lack of information about COVID‐19, changing policies, misinformation and concerns about PPE shortages, stigmatization by the public and concerns of infecting families (Preti et al., 2020). Besides the challenge to keep patients and their families safe, the emotional challenges of nurses included fear, anxiety, exhaustion, frustration, guilt and loneliness (Nelson et al., 2021). Nurses also experienced acute stress and depressive symptoms (Shechter et al., 2020). Anxieties appear to be limited to the acute phase of pandemic exposure, but life stress and burnout can be ongoing after the pandemic (Preti et al., 2020).

Individuals' reactions and coping strategies differ when they are exposed to stressful incidents and events. While some react negatively to stressful and traumatic situations, resulting in psychological distress, others quickly overcome the negative mental state and return to their normal lives (LeDoux & Gorman, 2001). This may empower people who can recover and resume their lives, which is referred as psychological resilience (Slavich et al., 2021). During the COVID‐19 pandemic, resilience strategies can help to alleviate emotional and psychological harm and pave the way for recovery and personal growth (Greenberg et al., 2020).

Self‐esteem, or self‐worth, may enhance the psychological resilience process (Barf et al., 2007). Studies conducted among nurses working with COVID‐19 revealed that resilience is affected by different personal characteristics such as self‐esteem (Ou et al., 2020). Studies on adolescence have shown self‐esteem as a statistically significant predator of resilience (Karatas & Cakar, 2011). If self‐esteem is dysfunctional may result in poor psychological outcomes, including poor social interactions and confidence to handle situations (Neff, 2011).

Another main aspect, which is prevalent during the pandemic and indirectly influences resilience is self‐compassion. Self‐compassion means being concerned and compassionate to oneself when confronted with difficulty or adversity (Neff, 2003). Self‐compassionate people are less likely to develop negative self‐evaluations and always have a sense of worth, which inter‐reflecting on self‐esteem (Leary et al., 2007). Research shows that self‐compassion significantly influences the behaviours and psychosocial skills in the general population, such as happiness, positive self‐evaluation and greater social connectedness (Gutiérrez‐Hernández et al., 2021).

In Qatar, few studies have been conducted on nurses during the COVID‐19 pandemic to evaluate their resilience in relation to self‐esteem and compassion aspects. This study aimed (1) to explore the self‐esteem and self‐compassion and psychological resilience of staff nurses and (2) to determine the association between the socio‐demographic data and study variables. The study will assist in exploring the influence of the pandemic in developing the nurses' resilience, self‐compassion and self‐esteem.

### 1.1 Theoretical framework

The compensatory model (Wang et al., 2021) and Shame Resilience Theory (Greene, 2017) underpinned this study.

As per the Shame Resilience Theory, self‐compassion can lead people to understand and calm their inner critic, which is key to living a brave life and helps to develop inner resilience (Hernandez & Mendoza, 2011). Initially, nurses during COVID‐19 will go through fear, self‐blame and disconnection from society, but as their experience is progressing, they start to establish meaningful connections with people (such as families and colleagues), which makes them strong and empathetical. The shame resilience continuum will progress from the fear zone to the growth zone, which helps them to achieve self‐compassion and ultimately resilience (Brown, 2006).

The compensatory model outlined the relationship between self‐esteem and resilience. The model identified self‐esteem as a compensatory factor to increase the resilience level. As per the model, resilient adults are the ones who learned from stressful experiences in a positive light even if they are suffering (Wang et al., 2021). As stated earlier, nurses who worked during COVID‐19 suffered from many stressors as a result of the pandemic over the three different waves, which may lead the nurses to learn from stressful experiences (COVID‐19) and develop self‐esteem and eventually become resilient.

## 2 METHODS

### 2.1 Design

A descriptive, cross‐sectional research survey design was utilized. The study was conducted at the largest health organization in Qatar. This organization includes 14 health facilities, covering all the medical needs of Qatari residents and citizens. The organization employs more than 10,000 nursing staff working in different facilities.

### 2.2 Participants

The target population of the study were Registered Nurses working in the health organization in Qatar. The sample size was calculated based on the previous study's mean resilience score (66.91 ± 13.34) (Alameddine et al., 2021), with a target population of 10,000 nurses, and 95% confidence interval, the sample size was 268. Assuming the 12% non‐response rate, the final the sample size was calculated as 300.

The inclusion criteria of the study participants were health professional licensed staff nurses with a minimum of 1‐year experience.

### 2.3 Data collection

A structured questionnaire was used to collect the data. The first part of the questionnaire was the demographic data of the participants. The second part of the questionnaire consists of 3 validated scales including.

- Connor‐Davidson Resilience Scale is a five‐point Likert scale with 10 items to measure resilience ranging from 0 to 4 in which 0 indicates “not true at all” and 4 indicates “true nearly all the time”: A respondent's total score can range from 0 to 40. higher scores indicate higher resilience (Connor & Davidson, 2003). Reliability was analysed by calculating McDonald's omega coefficient, which yielded a value of 0.83 (Alarcón et al., 2020).
- Rosenberg Self‐Esteem Scale is a 4‐point Likert scale with 10 items, measuring both positive and negative feelings about self‐indicating global self‐worth. The scale ranges from 0 to 4 in which 0 indicates “strongly disagree “and 4 indicates “strongly agree” A respondent's total score can range from 10 to 40. Higher scores indicate higher self‐esteem in which 0–20 is considered as low, 21–33 is considered normal and 34 to 40 is considered as high (Rosenberg, 1965). Reliability has been confirmed throughout a number of studies across a variety of cultures reporting alpha reliabilities ranging from 0.72 up to 0.90 (Gray‐Little et al., 1997).
- Self‐Compassion Scale‐Short Form (SCS‐SF) is a five‐point Likert scale with 12 items ranging from 1 to 5. 1 indicates “almost never” and 5 indicates” almost always”. A respondent's total score can range from 12 to 60. Higher scores indicate higher self‐compassion. The scale also addresses the subdomains such as Self‐Kindness, Self‐Judgement, Common Humanity, Isolation, Mindfulness and Over‐identified each item contains 2 questions and a score range from 1 to 10 (Raes et al., 2011). The scale suggested good reliability encompassing Cronbach's *α* coefficient (0.84) and test–retest reliability (0.89, in the 2‐week interval) (Meng et al., 2019).

### 2.4 Study procedures

The anonymized data were collected through online surveys using Microsoft forms. The survey was distributed widely via the hospital staff nurses’ email in which the participants were receiving the survey link along with the information sheet. A reminder message was sent every two weeks to increase the response rate. The researchers had no formal relationship with the participating nurse. The survey was opened in January 2022 when the state was facing the third wave of COVID‐19 pandemic as per the Ministry of Public Health, Qatar.

### 2.5 Data analysis

A total of 300 subjects were collected on January 2022. Descriptive statistics were used to summarize and determine the sample characteristics and distribution of participants’ data. The score was calculated for self‐esteem, self‐compassion and resilience to add the responses of nurses. The scores (self‐esteem, self‐compassion and resilience) were reported with mean and standard deviation (SD); categorical data were summarized using frequencies and proportions. Pearson correlation was calculated between the scores of self‐esteem, self‐compassion and resilience. Quantitative data between two or more independent groups were analysed using the unpaired *t* ANOVA test as appropriate. All *p*‐values presented were two‐tailed, and *p*‐values <0.05 were considered as statistically significant. All Statistical analyses were done using the statistical packages STATA 17.0 and Epi‐info (Armonk and Epi‐info Centers for Disease Control and Prevention, Atlanta, GA).

## 3 RESULTS

### 3.1 Sample characteristics

A total of 300 nurses completed the survey, with an average age of 38.2 years old (SD = 7.2), of whom the majority were females (76%), married (76%) and staff Nurses (74%). Third, of the Nurses were having more than 11 years of experience (35%), 26.3% 1–3 years, 25.7% 6 to 10 years. The majority of participants were graduate Registered Nurses (72.3%), 14% were charge nurses, 5% were nurse educators, 6% were head nurses, and 2% were executive and directors of nursing. The majority (60.3%) of the nurses were assigned to COVID facilities prior to the study. Table 1 shows the socio‐demographic data of the sample.

**TABLE 1:** Socio‐demographic characteristics of the participants (*n* = 300).

Factor	Level	Value (*N* = 300)
Age in years, mean (SD)		38.2 (7.2)
Gender	Male	72 (24.0%)
	Female	228 (76.0%)
Marital status	Single	65 (21.7%)
	Married	228 (76.0%)
	Divorce	3 (1.0%)
	Widowed	4 (1.3%)
Educational qualification	Diploma nursing	29 (9.7%)
	BSN	222 (74.0%)
	Master's degree and above	49 (16.3%)
Years of experience in HMC	1–3 years	79 (26.3%)
	4–5 years	39 (13.0%)
	6–10 years	77 (25.7%)
	11 & above	105 (35.0%)
Designation	Charge nurse	42 (14.0%)
	Executive/director of nursing	8 (2.7%)
	Graduate registered nurse	217 (72.3%)
	Head nurse	18 (6.0%)
	Nurse educator/researcher	15 (5.0%)
COVID‐19 deployment status	Assigned before	181 (60.3%)
	currently working	65 (21.7%)
	Never assigned	54 (18%)

**SCATTER PLOT 1:**
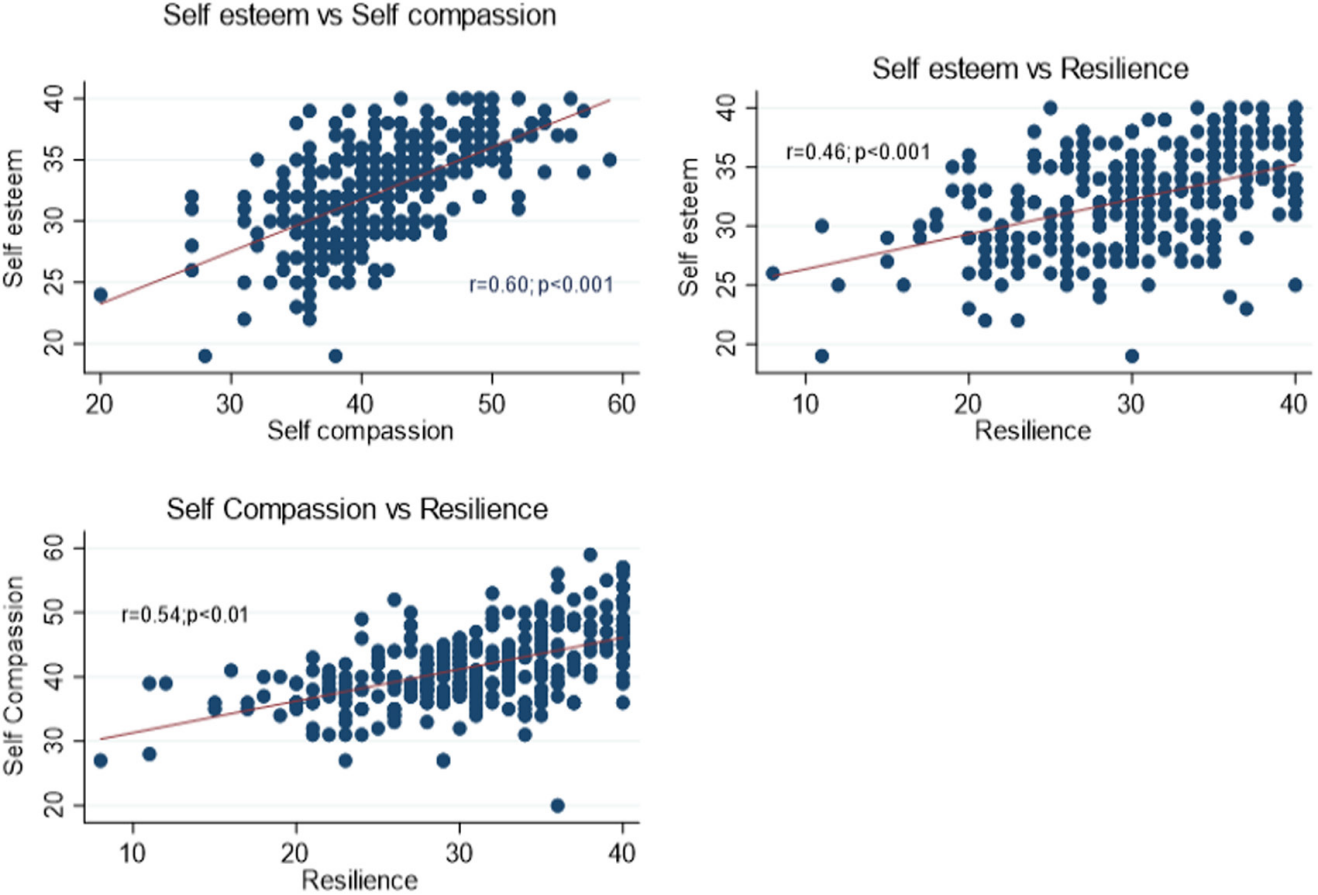
Correlation between self‐esteem, self‐compassion and resilience score.

### 3.2 Resilience, self‐esteem and self‐compassion of study participants

The average resilience scores of the participants were 30.2 ± 6.5. The mean scores of Self‐Esteem Scale scores of nurses were estimated to be, 32.3 ± 4.2, which comes under the normal range of 21–33, indicating nurses have normal self‐esteem.

The average self‐compassion score in nurses was 41.3 ± 5.9. The results showed that the highest self‐compassion subdomain is mindfulness (7.9 ± 1.55) followed by kindness (7.3 ± 1.55), judgement (6.8 ± 2.01), humanity (6.6 ± 1.47), over‐identified (6.5 ± 1.91) and lastly is isolation (6.2 ± 1.99). Table 2 represents the resilience, self‐esteem and self‐compassion of study participants.

**TABLE 2:** Descriptive statistics study variables (*n* = 300).

Status	Mean	SD	Median; (IQR) range
Resilience	30.2	6.48	40.0 (37.5, 45.0)
Self‐esteem	32.3	4.16	32.0 (29.0, 35.0)
Self‐compassion	41.3	5.91	30.0 (26.0, 35.0)
Kindness	7.3	1.55	7.0 (6.0, 8.0)
Judgement	6.8	2.01	7.0 (6.0, 8.0)
Humanity	6.6	1.47	7.0 (6.0, 8.0)
Isolation	6.2	1.99	6.0 (5.0, 8.0)
Mindfulness	7.9	1.55	8.0 (7.0, 9.0)
Over‐identified	6.5	1.91	6.0 (5.0, 8.0)

### 3.3 Correlations between resilience, self‐esteem and self‐compassion

All study variables were significantly positively correlated with each other. However, the correlation between self‐esteem and resilience (correlation coefficient(*r*) = 0.46; *p* < 0.001), self‐compassion and resilience (*r* = 0.54; *p* < 0.001) were moderate and self‐esteem and self‐compassion(*r* = 0.60; *p* < 0.001) scores were strongly correlated (*r* = 0.60; *p* < 0.001). Scatter plot 1 represents the correlation between the study variables.

### 3.4 Association of socio‐demographic variables with study variables

Education and job position were significantly associated with self‐esteem and resilience. In terms of self‐esteem, a master's and/or PhD 34.2 ± 4.0 had a higher score compared to a Diploma in nursing 32.1 ± 3.7 and a Bachelor of science in nursing 31.9 ± 4.2; *p* = 0.002. The resilience score was higher than those who completed Master 32.6 ± 4.6 and PhD compared to Bachelor of Science in Nursing 30.0 ± 6.5 and Diploma Nurses 28.4 ± 7.1; *p* = 0.009.

The Nursing Director or Executive and Head Nurses were having significantly higher self‐esteem scores 35.5 ± 4.9 and 35.1 ± 3.1, respectively, compared to Charge Nurses 33.5 ± 3.9, Nurse educator/*r*‐0.46; *p* < 0.001 researcher 33.5 ± 4.4 and graduate Registered Nurses 31.7 ± 4.1; *p* < 0.001. in terms of resilience, nursing director or executive (32.4 ± 7.1), head nurse (32.9 ± 4.9) and charge nurses (32.1 ± 5.8) had a higher score as compared to nurse educator/ researcher 28.1 ± 5.3 and Graduate Registered Nurses 29.7 ± 6.7; *p* < 0.032.

No association was found in terms of age, gender, marital status, or experience in the organization, assigned to COVID facilities with self‐esteem, self‐compassion and resilience scores. Table 3 shows the association between demographic variables and self‐esteem, resilience and self‐compassion.

**TABLE 3:** Association between demographics and self‐esteem, resilience and self‐compassion.

Factor	*N*	Self‐esteem, mean (SD)	Self‐compassion, mean (SD)	Resilience score, mean (SD)
Age in years				
20–30	36	31.3 (4.3)	40.3 (5.8)	29.3 (7.3)
31–40	173	32.2 (4.2)	41.2 (6.2)	30.5 (6.3)
41–50	66	32.8 (4.0)	42.0 (5.7)	29.8 (7.1)
50 & above	25	33.0 (4.0)	41.0 (4.4)	31.4 (4.9)
*p*‐value		0.25	0.55	0.56
Gender				
Male	72	32.9 (3.9)	41.4 (5.8)	31.2 (6.8)
Female	228	32.1 (4.3)	41.2 (6.0)	29.9 (6.4)
*p*‐value		0.17	0.80	0.14
Marital status				
Single	65	32.0 (4.4)	41.4 (6.2)	30.8 (7.0)
Married	228	32.4 (4.1)	41.3 (5.7)	30.0 (6.4)
Divorce/widowed	7	33.0 (5.2)	40.1 (10.4)	33.6 (4.6)
*p*‐value		0.74	0.86	0.26
Education status				
Diploma nursing	29	32.1 (3.7)	42.1 (6.0)	28.4 (7.1)
BSN	222	31.9 (4.2)	41.0 (5.6)	30.0 (6.5)
Master's degree and above	49	34.2 (4.0)	42.2 (7.2)	32.6 (5.6)
*p*‐value		0.002	0.30	0.009
Experience in HMC				
1–3 years	79	31.8 (4.3)	41.2 (6.0)	29.7 (7.4)
4–5 years	39	32.6 (3.5)	41.4 (6.2)	32.1 (5.2)
6–10 years	77	32.4 (4.4)	40.7 (5.9)	29.9 (6.4)
11 & above	105	32.6 (4.1)	41.7 (5.7)	30.2 (6.1)
*p*‐value		0.64	0.74	0.29
Job position				
Charge nurse	42	33.5 (3.9)	42.1 (4.8)	32.1 (5.8)
Executive/director of nursing	8	35.5 (4.9)	43.5 (7.3)	32.4 (7.1)
Graduate registered nurses	217	31.7 (4.1)	41.1 (6.0)	29.7 (6.7)
Head nurse	18	35.1 (3.1)	42.8 (6.7)	32.9 (4.9)
Nurse educator/researcher	15	33.5 (4.4)	39.3 (4.9)	28.1 (5.3)
*p*‐value		<0.001	0.28	0.032
COVID‐19 deployment status				
Assigned before	181	32.2 (4.2)	41.5 (6.1)	30.1 (6.6)
Currently working	65	32.9 (3.7)	41.6 (6.0)	31.4 (5.5)
Never assigned	54	32.0 (4.5)	40.4 (5.1)	29.2 (7.0)

## 4 DISCUSSION

The COVID‐19 pandemic has a substantial impact on nurses' psychological resilience, self‐esteem and self‐compassion. This study sought to examine the level of resilience among nurses working during a pandemic and the correlation with self‐esteem and self‐compassion. The study showed that resilience has a statistically significant correlation with self‐esteem and self‐compassion. The education level and job positions of nurses was associated with higher resilience and self‐esteem level.

### 4.1 Nurses' resilience

The participants in the current study scored a higher resilience score (30.2 ± 6.5) than participants in previous literature. A previous study conducted among healthcare professionals (including nurses) in Turkey showed that the resilience score was low (18.43 ± 4.2.) after the first wave of COVID‐19 (Bozdağ & Ergün, 2020). However, the current study was conducted after the third wave of COVID‐19 pandemic. The high resilience score among nurses during the third wave of pandemic in the current study could be explained by the previous experience during the first and second waves. The nurses in the current study may have learned from the first and second waves of the pandemic and they have developed a good experience in dealing with such circumstances (COVID‐19) leading to a higher resilience. Exposure to previous epidemics could lead the nurses to be more conversant with large outbreaks and the public health measures that are implemented as a result. Resilience building was slowly progressing with the pandemic (Roberts et al., 2021). The hardships faced by nurses during the pandemic made them to be strong and develop high resilience (Roberts et al., 2021). Improving nurses' resilience will be flourished after stressful events, which will help them to adapt quickly and reduce the long‐term effects of negative psychological experiences (Shechter et al., 2020).

### 4.2 Nurses' self‐esteem

The nurses in the current study reported relatively high self‐esteem (32.3 ± 4.2). Although the trajectory of self‐esteem was not measured in the current study, the fact that this study was measuring the level of self‐esteem after the third wave nurses may have developed their self‐esteem after their exposure to two previous waves. Self‐esteem changes systematically across time, it is relatively stable around its trait level in response to environmental conditions (Reitz, 2022).

The result is consistent with a study conducted on determinants of self‐esteem among healthcare professionals working in India during COVID‐19 pandemic that reported a high level of self‐esteem among the majority (70%) of participants (Radhakrishnan et al., 2021). The authors suggested that even though nurses have their own anxieties, they continue to deliver professional duties, which strengthen their level of Self‐esteem (Radhakrishnan et al., 2021).

Self‐esteem is an important safety factor protecting nurses from psychological harm during the pandemic (Reverté‐Villarroya et al., 2021). It is considered the strongest source of hope among health workers affected by the pandemic (Ramaci et al., 2020). As nurses in the study have expressed normal self‐esteem it is evidenced that they overcome psychological harm and become hopeful in dealing with the circumstances.

### 4.3 Nurses' self‐compassion

The nurses in the current study show relatively high scores in regard to self‐compassion. This is indicating that nurses are aware of their self‐empathy and coping with difficult circumstances (Harvey & Boynton, 2021). There is scientific evidence that self‐compassion is associated with well‐being, emotional intelligence, feelings of competence, happiness, optimism and wisdom leading to fundamental improvement of care provided by nurses (Gracia‐Gracia & Oliván‐Blázquez, 2017).

The self‐compassion level in the current study is higher than the score reported among nurses in Spain during the COVID‐19 pandemic where reported self‐compassion with an average of 19.8 ± 4 0.4 (Gutiérrez‐Hernández et al., 2021). Authors highlighted that self‐compassion plays an important role as a protective factor for nurses during COVID‐19. There is empirical evidence that positive levels of self‐compassion improve mental health and lessen the negative consequences of stressful events that may affect nurses (Cleare et al., 2019). A recent study on the self‐compassion of nurses during a pandemic reveals that self‐compassion may contribute to life satisfaction via positive coping (Lluch‐Sanz et al., 2022).

### 4.4 Correlations between resilience self‐esteem and self‐compassion

Self‐esteem and resilience were significantly positively correlated with each other in the current study. This result is consistent with a previous study on resilience and self‐esteem in healthcare workers of a COVID‐19 hospital in Bosnia, which concluded that resilience and self‐esteem were statistically significantly correlated with one another (Franjić et al., 2021). The author highlighted that resilience and self‐esteem reduced the effect of negative life events on positive social adjustments (Franjić et al., 2021). A high level of self‐esteem not only helps individuals to develop themselves despite existing challenges but also protects increases resiliency (Arslan, 2016). The strong correlation between self‐esteem and resilience can be attributed to the fact that those with a higher level of self‐esteem are optimistic and persistent individuals with a strong will for success (Long, 1970).

Resilience and self‐compassion were also significantly correlated in the current study. Nurses who are more kind to themselves, prevent self‐judgement and are more self‐compassionate, have a higher level of resilience (Kotera et al., 2021). This result is similar to the result of a study conducted among health professionals who identified that resilience and self‐compassion were positively correlated with each other (Ruiz‐Fernández et al., 2021). People with the stressful event who had high self‐compassion showed fewer symptoms of anxiety and depression and have higher resilience and quality of life (Raque‐Bogdan et al., 2011).

Self‐esteem and self‐compassion are other variables, which are significantly and positively correlated in the current study. The result is consistent with a study among nurses whose self‐compassion and self‐esteem was moderately related (Heffernan et al., 2010). The study found that self‐compassionate persons are less fearful of failure and perceived themselves as more competent and confident, which increases self‐esteem. Increasing self‐compassion will promote better mental health in the pandemic situation (Gutiérrez‐Hernández et al., 2021), which may increase self‐esteem (Ruiz‐Fernández et al., 2021) and resilience (Mert & Aker, 2019).

### 4.5 Association of demographics of participants with study variables

Nurse education and job role (senior roles) level have a statistically significant influence on self‐esteem and resilience scores. It is worth noting that as part of the career pathway in the organization (study setting) that the senior‐level in nursing position must have at least a Master's degree or above to hold such a position. Thus, the main characteristic of such senior positions is their educational level. This is evident in the result of the participants that the education level result has a statistically significant association with self‐esteem and resilience. The roots of building high self‐esteem may lie in the educational level (Milisen et al., 2010). In a study conducted to identify the role of education on resilience, authors found that nurses with an associate degree had better levels of resilience, as they were thought to be using social resources in a much better way (Afshari et al., 2021). This is could be explained by two sides. Firstly, education will strengthen nurses' coping skills in stressful situations leading to more self‐confidence and resilience (Middleton et al., 2021). Education in nursing produces a concept of self as a nurse, self‐confidence and self‐worth (Ghezelbash et al., 2015).

Secondly, education allows nurses to exercise reflection leading to be more aware of self‐compassion and then becoming more resilient. The practice of self‐compassion among highly educated nurses is healing and regenerating over time. Supporting nursing education in times of crisis will build their resilience (Neff et al., 2007). Managers should consider incorporating continuous educational programs that can increase nurses' self‐esteem and resilience. In addition, incentives such as economic rewards for nurses' who participate in continuous education may work (Manomenidis et al., 2018).

### 4.6 Limitations and recommendation

Despite the rigour with which the study was conducted, some limitations need to be acknowledged. Firstly, this study was conducted using convenience sampling from different hospitals, which are all located in Qatar, which limits generalization to other institutions.

Secondly, the study used an online questionnaire, which may have introduced some reporting bias. Self‐reporting questionnaires may introduce bias due to social desirability, which is the tendency of participants to present a more positive image of themselves (Johnson & Onwuegbuzie, 2004). Participants may assume the information they report (self‐deception) or may fabricate responses to conform to socially acceptable values or avoid criticism (Logan et al., 2008). Questions that are socially sensitive are most likely to elicit socially desirable responses (King & Bruner, 2000).

Initiate psychological interventions in frontline nurses to refine best practices and improve their Resilience in managing the psychological impact of future disasters. Further studies need to be done qualitatively to understand the circumstances that contributed to develop resilience self‐esteem and compassion.

## 5 CONCLUSION

Working during COVID‐19 imposed many challenges on nurses such as workload and stress. However, the different waves of the pandemic may increase the resilience, self‐esteem and self‐compassion of nurses. Their previous experiences in handling difficult situations during the pandemic may make the nurses become more confident in dealing with stressful situations and working under pressure. The education level had a statistically significant influence on nurses' higher resilience, self‐esteem and self‐compassion. More attention should be paid to junior‐level nurses as they are more vulnerable to the negative impact of the pandemic. However, a healthcare organization should monitor and explore the impact of such a crisis on a nurse's mental well‐being. The organization should engage with nurses and listen to their voices and opinions to be prepared for any future crisis.

## AUTHOR CONTRIBUTIONS

George Vellaramcheril Joy, Albara Mohammad Ali Alomari, Kalpana Singh, Nesiya Hassan, Kamaruddeen Mannethodi, Jibin Kunjavara and Badriya Al Lenjawi participated sufficiently in the work to take public responsibility for appropriate portions of the content.

## FUNDING INFORMATION

Open Access funding was provided by the Qatar National Library.

## CONFLICT OF INTEREST STATEMENT

The authors declare no conflicts of interest.

## ETHICS STATEMENT

Institutional Review Board (IRB) approval (MRC‐ 01‐ 21‐ 723) was obtained from the Medical Research Centre (MRC). The questionnaires were emailed to all staff who were working in 14 health facilities. Implied consent was used, where staff could refuse to participate in the survey by not returning their answers (O'Neill, 2003). No identifiable information was obtained, and participants were informed that their participation was voluntary.

## Data Availability

Data available on request due to privacy/ethical restrictions.
